# High-pressure, high-temperature molecular doping of nanodiamond

**DOI:** 10.1126/sciadv.aau6073

**Published:** 2019-05-03

**Authors:** M. J. Crane, A. Petrone, R. A. Beck, M. B. Lim, X. Zhou, X. Li, R. M. Stroud, P. J. Pauzauskie

**Affiliations:** 1 Department of Chemical Engineering, University of Washington, Seattle, WA 98195-1750, USA.; 2 Department of Chemistry, University of Washington, Seattle, WA 98195-1700, USA.; 3 Department of Materials Science and Engineering, University of Washington, Seattle, WA 98195-2120, USA.; 4 Materials Science and Technology Division, Naval Research Laboratory, Washington, DC 20375, USA.; 5 Physical and Computational Sciences Directorate, Pacific Northwest National Laboratory, Richland, WA 99352, USA.

## Abstract

The development of color centers in diamond as the basis for emerging quantum technologies has been limited by the need for ion implantation to create the appropriate defects. We present a versatile method to dope diamond without ion implantation by synthesis of a doped amorphous carbon precursor and transformation at high temperatures and high pressures. To explore this bottom-up method for color center generation, we rationally create silicon vacancy defects in nanodiamond and investigate them for optical pressure metrology. In addition, we show that this process can generate noble gas defects within diamond from the typically inactive argon pressure medium, which may explain the hysteresis effects observed in other high-pressure experiments and the presence of noble gases in some meteoritic nanodiamonds. Our results illustrate a general method to produce color centers in diamond and may enable the controlled generation of designer defects.

## Teaser

We create defects for quantum technologies by using high temperatures and pressures to convert molecularly doped carbon to diamond.

## INTRODUCTION

The characterization and manipulation of dopants in diamond have generated a wide range of applications spanning quantum computing, sensing, and cryptography (*1*–*3*), biolabeling (*4*), determination of interstellar origin in meteoritic samples (*5*–*7*), and investigation of Earth’s mantle (*8*, *9*), due to the remarkable properties of the diamond host. The dense diamond lattice exhibits a negligible immune response, maintains a wide bandgap, and, notably, restricts heteroatom defect diffusion at temperatures far above the diamond-graphite phase line at atmospheric pressure. For example, a common defect in diamond, substitutional nitrogen, does not diffuse at temperatures below 2000°C (*10*). In quantum sensing applications, this low diffusion coefficient enables the reliable use of single defects such as the negatively charged nitrogen vacancy (NV^−^) center to optically measure local spatiotemporal variations, which modify the defect’s spin precession rate, without fear of color center migration over long time scales (*1*). Similar applications in quantum cryptography have been proposed for the negatively charged silicon divacancy (SiV^−^) center (*2*, *11*–*13*). Because diffusion doping is not practical in diamond at ambient pressure, ion implantation is typically used to incorporate heteroatomic defects. This process relies on Poisson statistics, Stopping Range of Ions in Matter (SRIM) calculations, and masking techniques to control color center generation in chemical vapor deposition (CVD) diamond substrates (*2*). However, ion implantation also creates extensive lattice damage, induces fragmentation of ions, and cannot deterministically produce polyatomic defects. Because of these challenges, progress in single-defect applications often occurs by bulk defect production with implantation, followed by confocal scanning searches for ideal color centers.

An alternative, bottom-up method for diamond synthesis is high-pressure, high-temperature (HPHT) equilibrium phase conversion (*14*–*17*). While HPHT processes have produced doped diamonds, the rational formation of heteroatomic defects has remained elusive (*16*). In addition, HPHT experiments, including diamond syntheses, conventionally use noble gas pressure media, which, if incorporated into the lattice, have been proposed as defects for quantum sensing. However, to date, noble gas defect formation, such as xenon-related dopants, has been restricted to ion implantation (*18*). Despite its nearly ubiquitous role in high-pressure experiments, noble gas pressure media are widely considered to be inert, and there are no studies regarding the conditions that lead to incorporation within the diamond lattice under HPHT conditions (*19*, *20*).

To overcome diamond’s low diffusion coefficient and to study the incorporation of noble gas dopants without ion implantation, we propose a bottom-up methodology to dope diamond by first synthesizing a doped amorphous carbon precursor and then converting it to diamond under HPHT conditions in a noble gas environment. This allows us to simultaneously integrate the desired dopant into carbon while it is thermodynamically stable with traditional synthetic chemistry techniques, rather than rely on ion implantation into a metastable diamond substrate, and investigate noble gas incorporation under HPHT conditions.

**Fig. 1:**
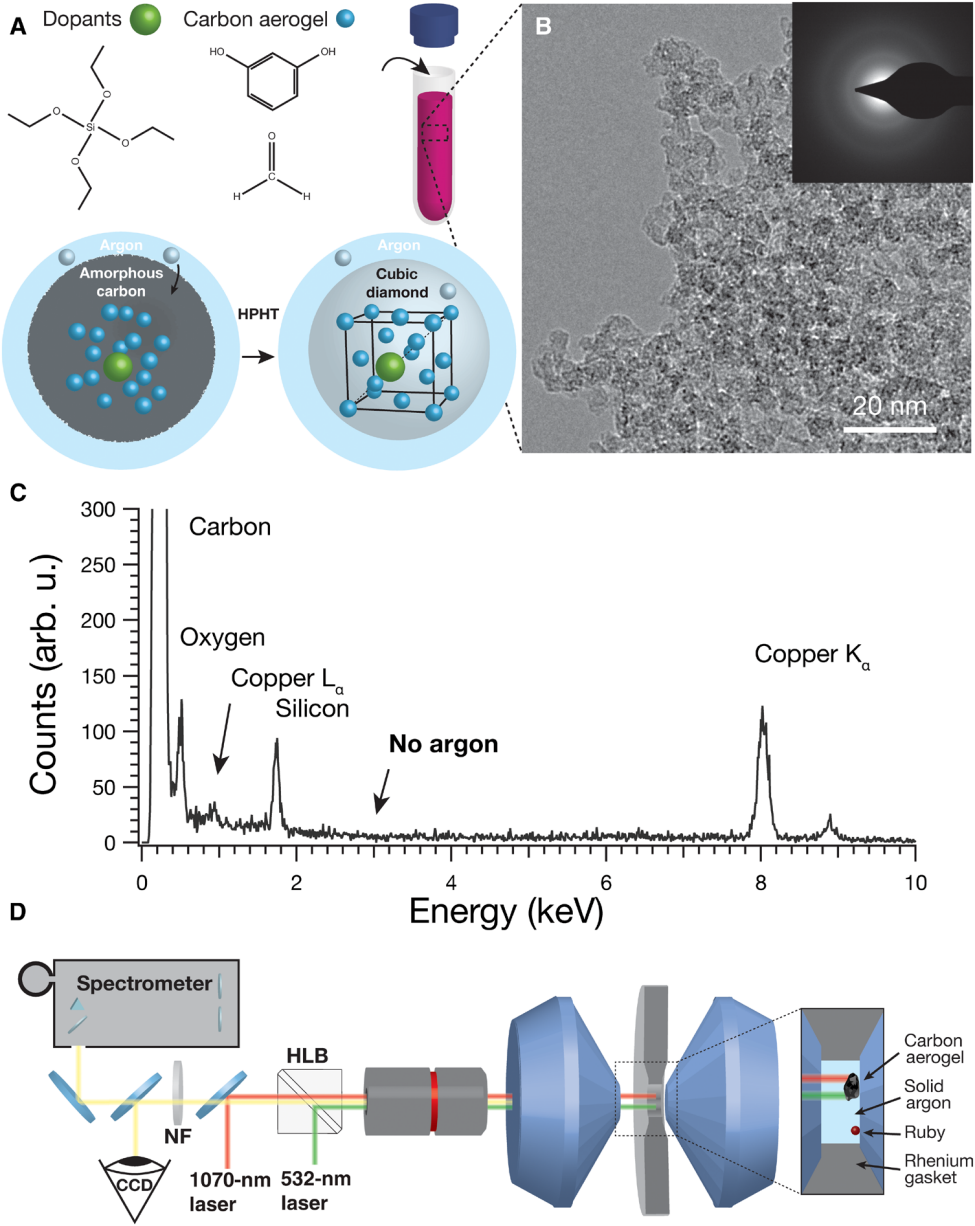
Carbon precursor doping mechanism and characterization. (**A**) Schematic representing the synthesis and doping of carbon aerogels, including BF-TEM image with SAED inset. To incorporate them within the carbon aerogel grains, we introduce dopants simultaneously with resorcinol and formaldehyde. Upon conversion to diamond at high pressure and high temperature, dopants remain inside the diamond lattice as color centers. (**B**) A TEM image with SAED of the doped carbon aerogel illustrates the carbon precursor morphology and lack of crystallinity. (**C**) EDS spectra of the carbon aerogel as synthesized only show the presence of carbon, silicon, and oxygen. Copper signal comes from the TEM grid. arb. u., arbitrary units. (**D**) Schematic showing a 1070-nm heating laser or polarized 532-nm Raman and photoluminescence (PL) laser focused into the pressurized DAC, which is loaded with a carbon aerogel precursor, ruby for pressure measurements, and solid argon pressure media, contained by a rhenium gasket. CCD, charge-coupled device; NF, notch filter; HLB, holographic beamsplitter cube.

Here, we probe these hypotheses by synthesizing a nanostructured carbon aerogel precursor with a controlled chemical composition and subjecting it to HPHT conditions in a laser-heated diamond anvil cell (DAC) with an argon pressure medium, as shown in Fig. 1 (*16*, *21*). Bright-field transmission electron microscopy (BF-TEM) and selected-area electron diffraction (SAED) in Fig. 1B demonstrate that the aerogel consists of 6.8 ± 1.9–nm-radius amorphous carbon grains. We tuned the chemical composition of the aerogel grains by adding tetraethylorthosilicate (TEOS) molecules directly to the mixture as it gelled (Fig. 1A). Energy-dispersive x-ray spectroscopy (EDS) confirmed that silicon dopants were incorporated throughout the carbon precursor material (Fig. 1C). To synthesize diamond, we placed the doped carbon precursor into a DAC and condensed solid argon within the high-pressure chamber to infiltrate the microstructure of the aerogel. We subsequently pressurized the cell above 20 GPa to thermodynamically favor diamond formation and drove grain growth by heating above 2000 K with a near-infrared (NIR) laser (Fig. 1C) (*14*).

## RESULTS

**Fig. 2:**
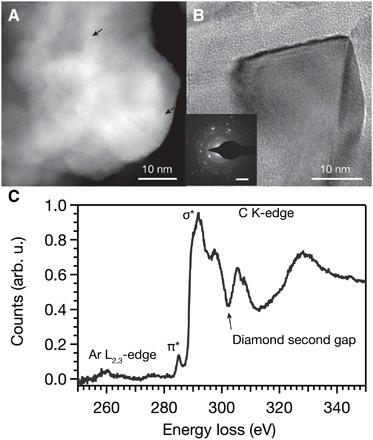
Structural characterization after HPHT synthesis. (**A** and **B**) High-angle annular dark-field scanning transmission electron microscopy (STEM-HAADF) and BF-TEM illustrate the microstructure of the recovered diamond material. Arrows in the STEM-HAADF image point out example impurity atoms. The identity of these atoms was not individually confirmed spectroscopically, but EDS mapping and Z-contrast indicate that most are silicon. The inset in (B) contains SAED, exhibiting 2.08-Å lattice spacings that correspond to diamond (111) planes. Figures S1 and S2 detail the *d*-spacing assignments and provide additional TEM images, respectively. (**C**) Power-law background subtracted STEM-EELS of the region displayed in (A) showing the argon-L_2,3_ edge and carbon-K edge.

To characterize the recovered material, we examined BF-TEM, SAED, and electron energy-loss spectroscopy (EELS). We found that the recovered material exhibited a network of predominately single-crystalline and occasionally polycrystalline nanoscale grains with crystallographic *d*-spacings corresponding to cubic diamond (Fig. 2B and fig. S1). The nanodiamond sizes ranged from 1 to 200 nm (fig. S2), indicating that significant carbon diffusion occurs during HPHT synthesis, which was likely enhanced by the high synthesis temperatures that surpass the melting point of argon at 20 GPa (1580 K) (*15*). The carbon-K edge EELS spectrum of pure diamond has a characteristic near-edge structure with a prominent σ* peak at 290 eV and a dip at 302.5 eV (*22*). The carbon-K edge spectrum of the recovered material contained both features, further indicating that the HPHT treatment formed cubic diamond, as well as a small pre-edge peak at 285 eV (Fig. 2 and fig. S10). This pre-edge feature corresponds to a π* excitation associated with sp^2^ carbon (*6*, *22*, *23*). As observed in previous HPHT and CVD experiments and in TEM images as a thin shell (fig. S2), this sp^2^ carbon likely stems from a nanodiamond surface reconstruction and incomplete sample heating due to the self-limiting absorption of amorphous carbon as it converts to diamond (*16*). As demonstrated in other nanodiamond syntheses, this surface sp^2^ carbon provides a useful moiety for surface functionalization, and removal by acid washing enables dispersion in a range of solvents (*4*, *24*). Low-loss EELS (fig. S4) data and Raman scattering (fig. S3) from the recovered material similarly indicate the prevalence of sp^3^ carbon in a diamond structure with a small amount of sp^2^ carbon (*25*). The combination of TEM, EELS, SAED, and Raman spectroscopy suggests almost complete conversion of the carbon precursor to diamond during this synthesis.

**Fig. 3:**
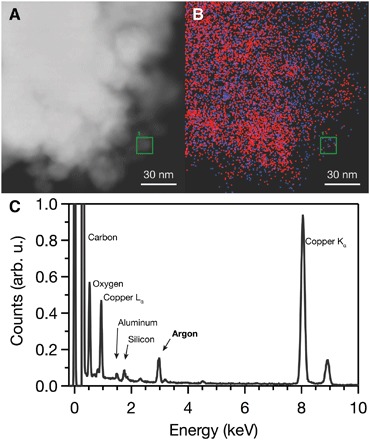
Dopant distribution mapping. (**A**) Wide-area STEM-HAADF image and (**B**) the corresponding STEM-EDS elemental mapping of argon (red) and silicon (blue) of the recovered TEOS-doped carbon aerogel. The green squares correspond to the field of view of Fig. 2A. (**C**) Extracted sum spectrum from the green square area of STEM-EDS spectrum image region showing the overall elemental composition. The small concentration of aluminum likely comes from trace amounts of ruby during laser heating, and the Cu peak is from the sample grid and STEM pole piece. The argon peak includes both K_α_ and K_β_.

EDS and EELS allow us to measure the chemical composition of the recovered nanodiamond and confirm the presence of dopants, including nitrogen, silicon, and argon. The *Z*-contrast of high-angle annular dark-field scanning transmission electron microscopy (STEM-HAADF) images identifies individual atoms and clusters (Fig. 2A). Combined, these data unambiguously demonstrate that silicon dopants added to the carbon aerogel precursor remain in and/or on the nanodiamond product after heating, despite significant grain growth. STEM-EDS elemental mapping reveals a predominantly homogeneous distribution of silicon and argon dopants throughout the diamond lattice (Fig. 3 and figs. S7 and S10). While most of the argon was distributed homogeneously, a few areas of higher argon concentration were observed in EDS maps of other particles. Individual argon atoms might incorporate as isolated defects resembling the neutral xenon defect, which is believed to occupy a split vacancy center along the <111> axis (*18*), and may be associated with twin boundaries. The areas of high concentration appear to be argon vesicles formed by closing of pores between aerogel precursor particles during the transformation to diamond. However, no segregation of silicon was observed. These data suggest that, during HPHT synthesis, silicon and argon atoms are trapped within the diamond lattice and that their presence is not due to adsorption or pressure-induced interfacial chemical bonding. The presence of argon within the recovered individual diamond particles, despite decompression to atmospheric pressure, transfer to a TEM grid, and analysis at ultrahigh vacuum conditions under a high-energy electron beam, further demonstrates robust incorporation within the diamond lattice, rather than surface adsorption.

We observed argon in all recovered samples synthesized at a range of pressures and temperatures from 20 to 25 GPa and 1800 to 3000 K. While other reports have demonstrated the effect of noble gas pressure media on samples at elevated pressures, such as partitioning of helium in SiO_2_ (*26*), this is the first confirmation of noble gas doping during a HPHT phase transition and stable incorporation upon decompression to atmospheric conditions (*27*). These results suggest that the aerogel structure allows argon to incorporate within its micropores during compression and that grain growth during laser heating traps these atoms within the lattice. For optoelectronic color center applications in diamond, this represents a new methodology for the incorporation of noble gas defects, e.g., xenon, for quantum computing and sensing (*18*). In addition, noble gas pressure media are almost exclusively used in HPHT experiments because they remain hydrostatic to high pressures and are chemically and physically inert (*28*). The incorporation of noble gas pressure media into materials under HPHT conditions challenges this view of complete inactivity and could explain hysteresis effects in previous DAC experiments (*29*–*31*). For example, the incorporation of noble gas into a sample under HPHT conditions in DAC experiments would increase its compressibility, thereby increasing the pressure required to drive a phase transition as pressure is ramped up and, depending on the kinetics of noble gas escape from the lattice, decreasing the pressure required to drive a phase transition as pressure is reduced. It could also provide an explanation for how noble gas atoms are incorporated into nanodiamonds in astrophysical environments (*5*).

**Fig. 4:**
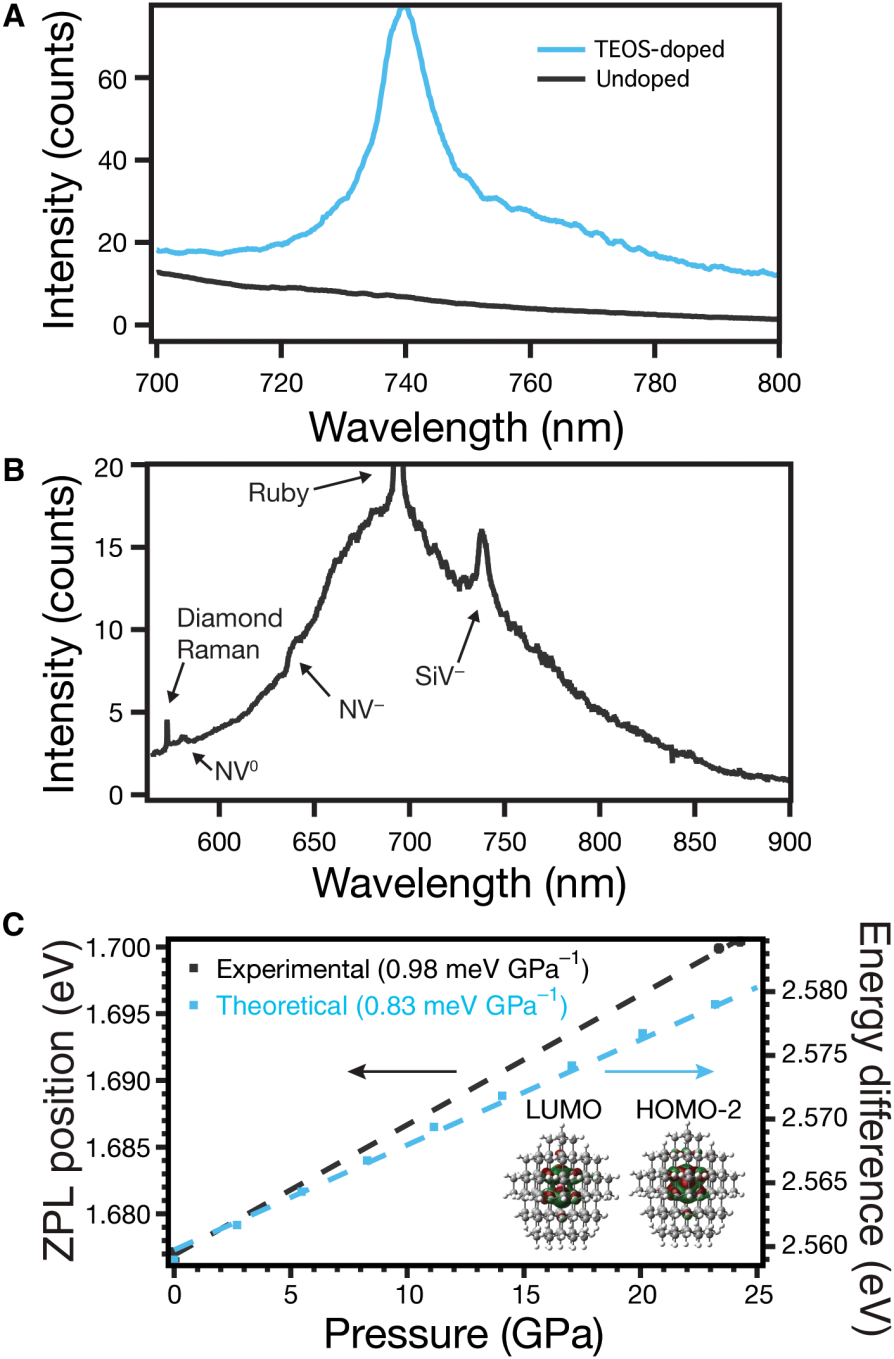
PL of color centers. (**A**) High-resolution PL spectra of the SiV^−^ region comparing HPHT TEOS-doped and undoped carbon aerogels. (**B**) PL and Raman scattering of recovered nanodiamond synthesized from the TEOS-doped carbon aerogel after depressurization and removal from the DAC. Labels denote diamond Raman scattering and NV^0^, NV^−^, and SiV^−^ color center ZPLs. (**C**) Experimental SiV^−^ ZPLs and the B3LYP/6-31G(d) average energy differences of the molecular orbitals, which exhibit largest contributions to the absorption peak responsible for the ZPL at different pressures. Error bars for both pressure and ZPL energy sit within the circular markers. The insets in (C) illustrate the contour plots (0.025 isodensity) of the LUMO (lowest unoccupied molecular orbital) (left) and the HOMO-2 (highest occupied molecular orbital) (right) molecular orbitals (the largest contribution; see table S1 and fig. S6) of a SiV^−^-containing nanodiamond (C_119_SiH_104_), oriented perpendicular to the diamond <1,1,1> axis, as modeled with DFT. White, gray, and pink atoms are hydrogen, carbon, and silicon, respectively.

The photoluminescence (PL) spectra of all the recovered material contain emission from NV centers (Fig. 4). The shoulders at 575 and 637 nm and the broad feature centered at 700 nm are uniquely characteristic of NV^0^ and NV^−^ zero phonon lines (ZPLs) and phonon side bands, which have been observed in multiple HPHT reports due to atmospheric N_2_ incorporation (*16*, *32*). PL signal from the argon is neither observed nor expected (*33*). However, all HPHT-treated silicon-doped carbon aerogel contain a peak at 739 nm, corresponding to the SiV^−^ color center, that is not present in undoped carbon aerogel (*34*, *35*). To confirm the SiV^−^ assignment, we collected pressure-dependent PL and conducted ab initio calculations to model the pressure dependence by fully simulating a nearly spherical C_119_SiH_104_ nanodiamond (~1.2 nm in diameter) containing a SiV^−^ defect under uniform hydrostatic pressure with density functional theory (DFT) using the Gaussian electronic structure package (*13*, *23*, *36*). The close agreement between experimental (0.98 ± 0.01 meV GPa^−1^) and theoretical (0.83 meV GPa^−1^) slopes from 0 to 25 GPa further confirms the presence of SiV^−^ in the diamond lattice (Fig. 4C). The high sensitivity of SiV^−^ to pressure and its narrow linewidth are comparable to the d-d transitions of Cr^3+^ in alumina (ruby), the nearly ubiquitous choice to measure pressure in DAC experiments. Extending the DFT simulation up to 140 GPa (fig. S5) demonstrates the viability of optical pressure metrology with SiV^−^ at high pressures. Ruby undergoes a phase transition at 94 GPa and 1300°C, making it unsuitable for the next-generation HPHT experiments, which have recently reached the terapascal range (*37*, *38*). On the other hand, diamond is the thermodynamically stable allotrope of carbon at all pressures above 1 GPa and temperatures until melting (*14*). The lack of phase transformation implies that SiV^−^-doped nanodiamond may succeed under conditions where ruby cannot be used.

## DISCUSSION

Contrary to traditional crystal growth, which predicts alloyed phase formation or clustering, the observation of SiV^−^ in the recovered nanodiamond confirms that dopants added to the carbon aerogel precursor produce homogeneously distributed color centers within the recovered nanodiamond. While a subset of dopants likely incorporate substitutionally, both SiV and NV are lower-energy states than their substitutional silicon and nitrogen counterparts due to lattice relaxation, as observed in Jahn-Teller distortions of the lattices (*39*, *40*). Unlike ion implantation, which requires annealing to drive vacancy diffusion to activate incorporated heteroatoms, optically active color centers form immediately upon HPHT conversion to diamond. Theoretical and experimental results show that, at these pressures, subnanometer sp^3^ carbon grains nucleate at each aerogel grain’s core and diamond growth with simultaneous Ostwald ripening continues radially (*15*, *41*, *42*). The production of homogeneously distributed defects suggests that, as the diamond lattice forms around the heteroatomic silicon and nitrogen atoms, the lowest-energy color center structure forms immediately. While silicon atoms have been doped into diamond before, the process involved ion implantation or complete chemical breakdown in plasma, which limited controllable heteroatom defect formation (*2*). This bottom-up approach illustrates the possibility of a new doping paradigm for diamond where molecular dopants can be designed with the precise heteroatomic stoichiometry and three-dimensional stereochemistry to create a wide range of polyatomic point defects.

The rational incorporation of silicon—by chemically doping the carbon precursor with TEOS—and argon—by using an argon pressure medium—into nanodiamond illustrates the potential impact of this doping methodology for doped nanodiamond applications (e.g., high-pressure metrology) without ion implantation. Rather than sequentially synthesizing diamond, implanting substitutional heteroatoms, annealing vacancy centers, and conducting confocal searches for color centers, the HPHT conversion of doped carbon can directly form color centers. For single-defect applications, this research opens the door to the incorporation of more complex defects into diamond with structures defined by the chemical dopant added into the carbon precursor. If diamond nucleates before dissociation of the dopant, then defects could be added with chemical precision limited only by molecular synthesis (*41*, *42*). In addition, multianvil presses can achieve sufficient conditions to drive diamond formation, potentially enabling industrial production of dispersible nanodiamonds with complex color centers (*24*). For extraterrestrial nanodiamonds, where dopants in diamond are used to fingerprint the presolar and interstellar environment, this demonstration unveils the DAC as a tool to study HPHT doping that could occur in astrophysical environments (*5*, *6*). Given the prevalence of noble gas pressure media, these results have broad implications for high-pressure experiments, where, to date, noble gasses have been considered inert (*26*, *28*).

## MATERIALS AND METHODS

We synthesized carbon aerogels by adding 0.104 g of resorcinol (Sigma-Aldrich, St. Louis, MO, USA), 0.141 ml of formaldehyde [37 weight % (wt %) methanol-stabilized aqueous solution; Sigma-Aldrich], and 0.112 ml of hydrochloric acid (37 wt %; Macron, Center Valley, PA, USA) to 3.747 ml of acetonitrile (EMD Millipore, Billerica, MA, USA) to achieve a molar ratio of resorcinol to formaldehyde, hydrochloric acid, and acetonitrile of 1:2, 8.4:1, and 1:76, respectively, and a final volume of 4 ml in a polycarbonate centrifuge tube to avoid contact with silicon-based glassware. For silicon-doped carbon aerogel, we serially diluted tetraethyl orthosilicate (98 wt %; Acros Organics) with acetonitrile to a final molar concentration of 4.5 × 10^−9^ M and used this acetonitrile solution to synthesize the silicon-doped carbon aerogel. This reaction yielded a nominal molar ratio of 2.03 × 10^−6^ silicon atoms per carbon atom, which was selected to produce approximately one silicon dopant per carbon aerogel grain. Additional Poisson calculations for the resulting nanodiamond grain sizes are provided in fig. S9. We transferred the solution to a Branson 1510R-DTH ultrasonic cleaner until the gel turned a light pink and solidified, typically 30 min. We then exchanged the acetonitrile solvent with ethanol four times over 5 days and dried the gel with supercritical CO_2_ in an autoclave (E3100, Quorum Technologies, Laughton, East Sussex, UK) to prevent pore collapse due to capillary pressure. Last, we pyrolyzed the gels at 1000°C in an inert atmosphere for 4 hours to remove oxygen moieties from the gel.

### HPHT synthesis

To achieve HPHT conditions, we used a laser-heated Boehler-Almax plate DAC with 0.300-mm-diameter culets. First, we dimpled a rhenium gasket from 250 to 30 μm—that is, we inserted a 250-μm-thick rhenium disc into the DAC and advanced the diamonds to reduce the gasket to a final thickness of 30 μm. Then, we drilled an 80-μm hole in the center of the gasket using an electronic discharge machine to form the walls of the high-pressure chamber. Next, we returned the gasket to the DAC and a Marzhauser Wetzlar nanomanipulator equipment with a tungsten probe to transfer approximately 15 grains of carbon aerogel on the order of 10 μm in diameter and finely ground ruby crystals into the DAC’s cavity. The carbon aerogel and ruby acted as a diamond precursor and a pressure monitor, respectively. To remove adsorbed species from the aerogel, we placed the DAC into a sealed chamber and flowed argon over it for at least 30 min. Afterward, we used liquid nitrogen to condense the flowing argon and subsequently tightened the DAC to trap liquid argon in the DAC’s chamber. By condensing liquid argon from a gaseous argon environment, we infiltrated the carbon aerogel precursor’s pores with gaseous and then liquid argon to maintain the aerogel pore structure as much as possible.

We laser-heated the DAC using a ytterbium-based, 1070-nm IPG Photonics YLR laser focused with a NIR-corrected Mitutoyo 50× objective [0.55 numerical aperture (NA)]. The laser rapidly heated the aerogel precursor, and we simultaneously collected Planck emission from 250 to 900 nm with a fiber-coupled Ocean Optics USB2000 spectrometer and 1000-nm dichroic mirror, which was calibrated with an Optics HL-2000. To calculate temperatures, we fit the wavelength- and intensity-corrected spectra with Wien’s Law and used this solution as the initial guess for a full nonlinear Planck’s solution in MATLAB. All measurements of the recovered carbon suggested that this HTHP procedure completely transformed the amorphous carbon to diamond, with graphitic particles only rarely observed in TEM. We collected Raman and PL using a home-built microscope composed of a Coherent compass 532-nm laser, a Mitutoyo 50× objective (0.55 NA), and a SpectraPro 500i with a Princeton Instruments liquid nitrogen–cooled detector. For color center PL, we acquired spectra at a power of 70 μW inside the DAC (through the diamond anvils) and 50 μW after recovery on a TEM grid with acquisition times of 5 s, which produced SiV^−^ counts between 2.5 and 37 kilocounts s^−1^. However, note that these samples have three-dimensional topology, making confocal scanning challenging. In addition, we measured some Raman spectra with a Renishaw inVia system equipped with a Leica DMIRBE inverted microscope and a 785-nm excitation source.

### Microscopy methods

Scanning transmission electron microscopy, including HAADF imaging, was performed with the Naval Research Laboratory (NRL) Nion UltraSTEM 200X operated at 60 kV. This instrument is equipped with a Gatan Enfinium ER energy-loss spectrometer and a Bruker XFlash100, 0.7sr EDS. The nominal probe conditions were a ~0.15-nm spot size with 50- to 100-pA current. For carbon core-loss fine-structure determination, a dispersion of 0.05 eV per channel was used, which provided an energy resolution of 0.4 eV, as measured by the full width at half maximum of the zero loss peak. The core-loss electron energy-loss data were acquired as multipass spectrum images, with spatial drift correction. Low-loss data were acquired as single-frame spectrum images. A power-law background was subtracted using a 10-eV window below the Ar-L_2,3_ edge. The EDS data from the diamonds were obtained as spectrum images. BF-TEM images and SAED patterns were taken at the University of Washington (UW) on a FEI Tecnai G2 F20 at an accelerating voltage of 200 kV. These images were used to quantify the nanodiamond diameters.

### Computational methods

Computational studies were performed using the Gaussian electronic structure package (*36*). A nearly spherical C_119_SiH_104_ nanodiamond (~1.2 nm in diameter) was constructed with a bulk face-centered cubic lattice parameter of *a* = 0.357 nm, and hydrogen atoms were used to passivate the surface carbon atoms and to saturate surface dangling bonds. Additional details are provided in the Supplementary Materials.

## Supplementary Material

http://advances.sciencemag.org/cgi/content/full/5/5/eaau6073/DC1

Download PDF

## SUPPLEMENTARY MATERIALS

Supplementary material for this article is available at http://advances.sciencemag.org/cgi/content/full/5/5/eaau6073/DC1 Additional computational details Fig. S1. SAED of the recovered nanodiamond material. Fig. S2. Additional TEM images of the recovered nanodiamond material. Fig. S3. Raman of the recovered nanodiamond material. Fig. S4. Low-energy EELS. Fig. S5. DFT modeling of the SiV^−^ defect in diamond under uniform hydrostatic pressure. Fig. S6. DFT modeling of the SiV^−^ excited states. Fig. S7. STEM-EDS composition maps. Fig. S8. Carbon-K edge scanning transmission x-ray microscopy of nanodiamond synthesized from undoped carbon aerogel on a lacey carbon TEM grid. Fig. S9. Poisson distribution of silicon incorporation per nanodiamond grain with varying size. Fig. S10. Integrated STEM-EEL spectrum image of the recovered silicon-doped carbon aerogel. Fig. S11. DFT modeling of surface capping scheme on SiV^−^ center excitations. Table S1. Time-dependent DFT transition energies and oscillator strengths. Table S2. Time-dependent DFT transition energies and oscillator strengths for ligand capping schemes. Table S3. DFT orbital differences for the ligand capping schemes. References (*43*–*60*)
