# Supplementary material for: High-pressure, high-temperature molecular doping of nanodiamond
Source: Sci Adv. 2019 May 3;5(5):eaau6073. doi: 10.1126/sciadv.aau6073 (PMC6499550; doi:10.1126/sciadv.aau6073)
Supplement: http://advances.sciencemag.org/cgi/content/full/5/5/eaau6073/DC1 [file supp_5_5_eaau6073__index.html]

Science Advances | Science Advances

## Supplementary Materials

**This PDF file includes:**

- Additional computational details
- Fig. S1. SAED of the recovered nanodiamond material.
- Fig. S2. Additional TEM images of the recovered nanodiamond material.
- Fig. S3. Raman of the recovered nanodiamond material.
- Fig. S4. Low-energy EELS.
- Fig. S5. DFT modeling of the SiV− defect in diamond under uniform hydrostatic pressure.
- Fig. S6. DFT modeling of the SiV− excited states.
- Fig. S7. STEM-EDS composition maps.
- Fig. S8. Carbon-K edge scanning transmission x-ray microscopy of nanodiamond synthesized from undoped carbon aerogel on a lacey carbon TEM grid.
- Fig. S9. Poisson distribution of silicon incorporation per nanodiamond grain with varying size.
- Fig. S10. Integrated STEM-EEL spectrum image of the recovered silicon-doped carbon aerogel.
- Fig. S11. DFT modeling of surface capping scheme on SiV− center excitations.
- Table S1. Time-dependent DFT transition energies and oscillator strengths.
- Table S2. Time-dependent DFT transition energies and oscillator strengths for ligand capping schemes.
- Table S3. DFT orbital differences for the ligand capping schemes.
- References (*43*–*60*)

Download PDF

**Files in this Data Supplement:**

- Adobe PDF - aau6073\_SM.pdf
