# Supplementary material for: High-pressure, high-temperature molecular doping of nanodiamond
Source: Sci Adv. 2019 May 3;5(5):eaau6073. doi: 10.1126/sciadv.aau6073 (PMC6499550; doi:10.1126/sciadv.aau6073)
Supplement: Download PDF [file aau6073_SM.pdf]

Science Advances
AAAS

advances.sciencemag.org/cgi/content/full/5/5/eaau6073/DC1

# Supplementary Materials for

## High-pressure, high-temperature molecular doping of nanodiamond

M. J. Crane, A. Petrone, R. A. Beck, M. B. Lim, X. Zhou, X. Li, R. M. Stroud, P. J. Pauzauskie*

*Corresponding author. Email: peterpz@uw.edu

Published 3 May 2019, *Sci. Adv.* **5**, eaau6073 (2019)
DOI: 10.1126/sciadv.aau6073

**This PDF file includes:**

Additional computational details
Fig. S1. SAED of the recovered nanodiamond material.
Fig. S2. Additional TEM images of the recovered nanodiamond material.
Fig. S3. Raman of the recovered nanodiamond material.
Fig. S4. Low-energy EELS.
Fig. S5. DFT modeling of the $SiV^-$ defect in diamond under uniform hydrostatic pressure.
Fig. S6. DFT modeling of the $SiV^-$ excited states.
Fig. S7. STEM-EDS composition maps.
Fig. S8. Carbon-K edge scanning transmission x-ray microscopy of nanodiamond synthesized from undoped carbon aerogel on a lacey carbon TEM grid.
Fig. S9. Poisson distribution of silicon incorporation per nanodiamond grain with varying size.
Fig. S10. Integrated STEM-EEL spectrum image of the recovered silicon-doped carbon aerogel.
Fig. S11. DFT modeling of surface capping scheme on $SiV^-$ center excitations.
Table S1. Time-dependent DFT transition energies and oscillator strengths.
Table S2. Time-dependent DFT transition energies and oscillator strengths for ligand capping schemes.
Table S3. DFT orbital differences for the ligand capping schemes.
References (*43–60*)

## Supplementary Materials

### Additional computational details

The SiV center in diamond consists of a silicon atom and a vacancy in a split-vacancy configuration (*43*, *44*). The SiV center was thus created by removing two carbon atoms near the center of the nanodiamond along the <1,1,1> axis of the model, and positioning the Silicon at the center of the resulting divacancy in a local $D_{3d}$ environment, as observed in previous computational studies (*38*, *41*, *42*, *45–47*). In this work, we focus on the reduced $SiV^-$ center, whose ground state has been shown to be a doublet (*48*).

The ground-state electronic structures were obtained by solving the Kohn-Sham equation using the hybrid Becke, 3-parameter, Lee-Yang-Parr (B3LYP) density functional (*49–51*) with a 6-31g(d) basis set. This theory level has been previously validated for the description of the electronic structure and optical properties of both pure and nitrogen vacancy doped nanodiamonds of the same dimensions (*52*).

Several models were prepared to investigate the effect of uniform hydrostatic pressure by shrinking the lattice parameter uniformly. The required change in the molar volume for a given applied pressure was obtained via the Vinet equation of states

$$P = \frac{3\,K_0(1-\chi)}{\chi^2} e^{\frac{3(K_0'-1)(1-\chi)}{2}}$$

with the parameters $K_0 = 446$ GPa and $K_0' = 3.0$, and $\chi = \sqrt[3]{V/V_0}$ , where $V_0$ is the molar volume at atmospheric condition (using the original bulk fcc lattice parameter) and $V$ is the

corresponding molar volume at pressure $P$ (*53*). $V$ was calculated for different pressure ranges determined by both experimental constraints and the limits of the validity of the equation of states, and the different structures were obtained by adjusting the lattice parameters accordingly (*53*).

The energy difference reported as a function of pressure in Fig. 4 and S1 is the average energy difference between the HOMO-1 and the LUMO, the HOMO-2 and the LUMO, and the HOMO-3 and the LUMO. These molecular orbitals exhibit largest contributions to the absorption peak, responsible for the ZPL, computed using the time dependent density functional theory (TD-DFT) within the linear-response framework (see Table S1) (*54–56*).

Some theoretical, DFT-based results have demonstrated that surface moieties can impact NV color center energy levels (*57*). However, the $SiV^-$ energy levels sit deeper in the diamond band gap, making them less susceptible to any influence from functional groups on the surface (*13*, *58*). To confirm this, we simulated the energy levels of a $SiV^-$ center in a nanodiamond at room pressure with a surface hydrogen replaced by either a carboxyl or a hydroxyl functional group (Table S2, fig. S11). The addition of these functional groups produced a very small absolute change (7.9 meV and 3.3 meV for carboxylate and hydroxide, respectively) in the predicted $SiV^-$ energy level difference, indicating their insensitivity to oxygen-containing moieties on the nanodiamond surface. The lack of electron density localized at these functional groups further underscores the independence of the $SiV^-$ energy levels to the oxygen-containing moieties at the nanodiamond surface. Moreover, any potential influence of surface functionalization on SiV-

center’s energy levels in nanodiamonds likely diminishes for larger nanodiamonds (>2 nm), including the majority our synthesized nanodiamonds (*13*, *35*).

**Supplementary Figures**

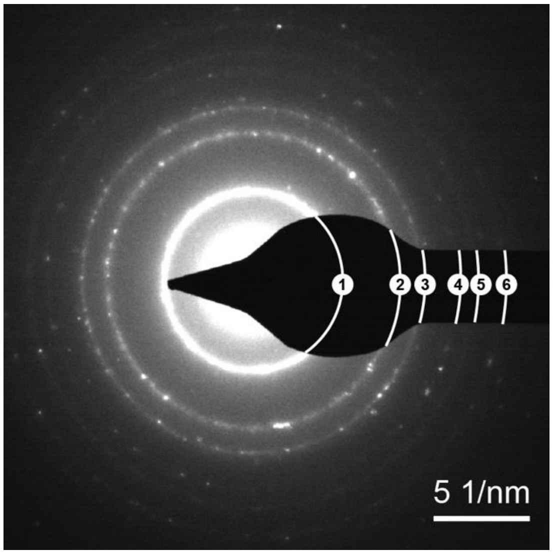


**Fig. S1. SAED of the recovered nanodiamond material.** The selected area electron diffraction of the recovered material after HPHT synthesis. The diffraction indexes to cubic diamond with d-spacings (and corresponding miller indices) of:  1, 2.08 Å (111); 2, 1.27 Å (220); 3, 1.08 Å (311); 4, 0.89 Å (311); 5, 0.83 Å (331); and 6, 0.74 Å (422). Note that we enhanced the contrast to identify the lower intensity d-spacings and search for foreign crystalline components.

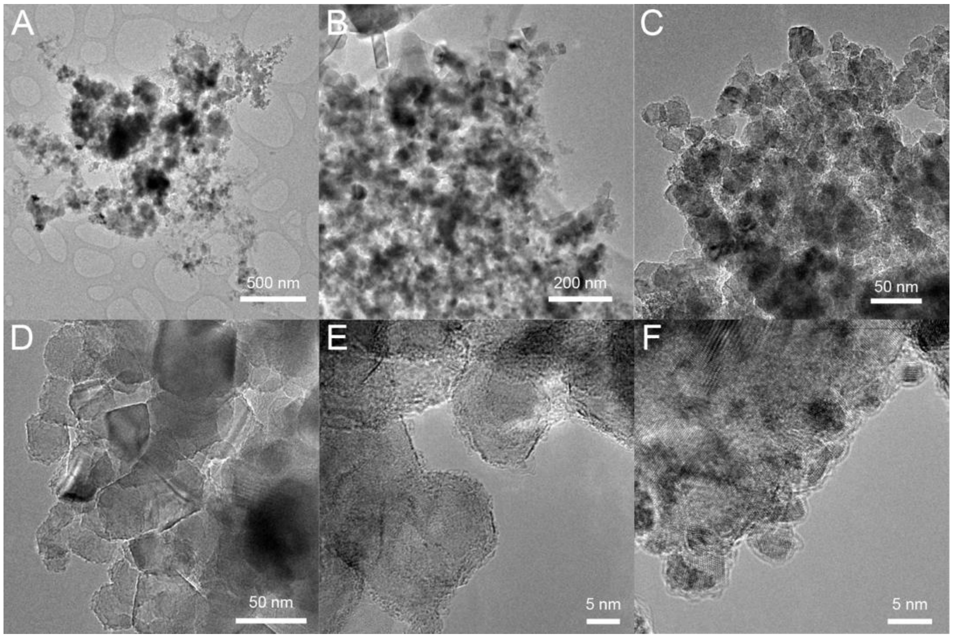


**Fig. S2. Additional TEM images of the recovered nanodiamond material.** Bright-field TEM images showing the morphology and structure of the carbon aerogel after HPHT processing. Panels (A) and (B) illustrate that, while there is ripening of the carbon grains during growth, much of the recovered material is composed of nanodiamond grains far below 50 nm in diameter, compared to amorphous carbon grains of 6.8 ± 1.8 nm before synthesis. For example, the predominately single crystalline, nanodiamond diameters are 14.0 ±5.8 nm and 29.2 ± 7.4 nm in panels (C) and (D), respectively. Poisson statistic estimates of doping for these sizes are provided in fig. S9. Grains with clear faceting and diameters below 10 nm can be seen in panels (E) and (F). The surface roughness is due to incomplete conversion due to self-limiting absorption as the amorphous carbon converts to diamond at high pressures and temperatures and surface reconstruction effects, as discussed in the main text.

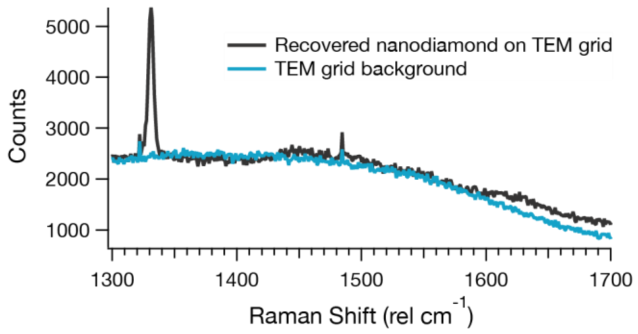


**Fig. S3. Raman of the recovered nanodiamond material.** Raman from the nanodiamond material shows a sharp peak at 1331.0 rel cm$^{-1}$, corresponding to cubic diamond and not to the D-band in disordered sp$^3$ carbon. In addition, there is no feature associated with the sp$^2$ carbon G-band, which occurs at approximately 1600 rel cm$^{-1}$ (*25*). The Raman laser wavelength was 785 nm.

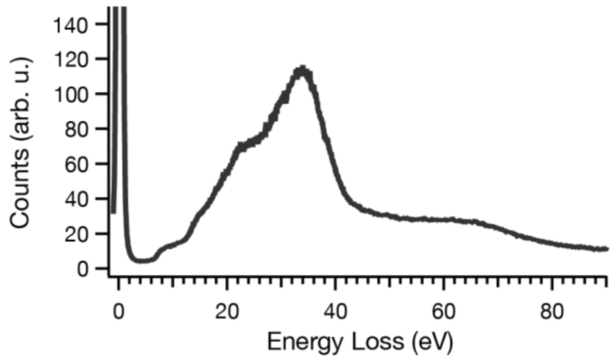


**Fig. S4. Low-energy EELS.** The strong peak at 34 eV corresponds to bulk plasmon in cubic diamond. The feature at 23 eV is likely due to sp$^2$ carbon at the particle surfaces. The broad feature at ~ 7 eV can be attributed to defects in diamond, and π-plasmon of the sp$^2$ surface carbon.

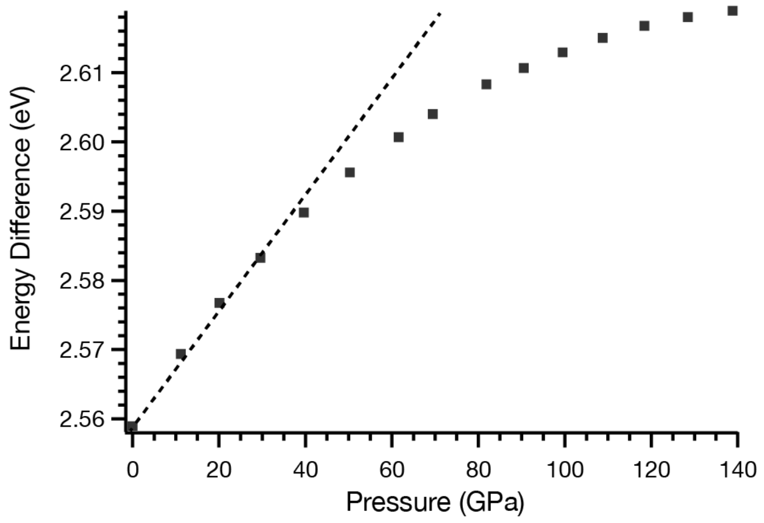


**Fig. S5. DFT modeling of the SiV$^{-}$ defect in diamond under uniform hydrostatic pressure.** Average energy differences of molecular orbitals that exhibit largest contributions to the absorption peak responsible for the ZPL at different pressures up to 100 GPa for the SiV$^{-}$ containing nanodiamond with diameter of ~1.2 nm ($C_{119}SiH_{104}$). The dashed line shows the linear fit used on the data up to ~30 GPa (shown in the main text within Fig. 4). Starting from ~40 GPa, a clear sublinear trend is observed. This trend has been observed before for the Raman frequency shift in diamonds under pressure (*53*) and has been qualitatively explained in terms of the antibond interaction in the interstitial region (*59*).

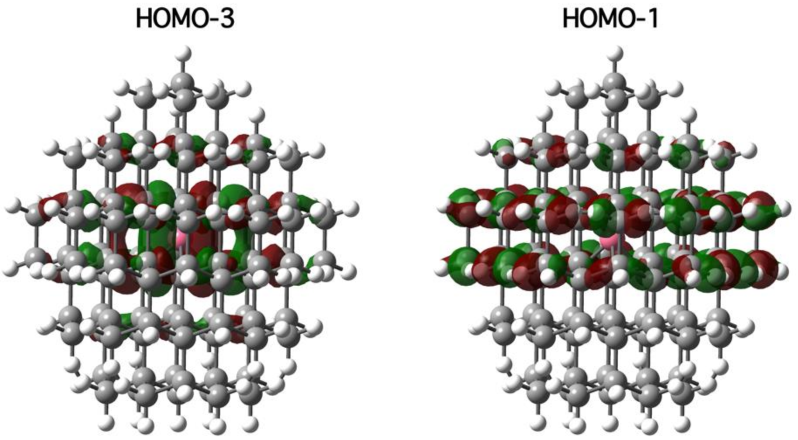


**Fig. S6. DFT modeling of the SiV⁻ excited states.** The B3LYP/6-31G(d) contour plots (0.025 isodensity) of the HOMO-3 and HOMO-1 for the $SiV^-$ containing nanodiamond with diameter of ~1.2 nm ($C_{119}SiH_{104}$).

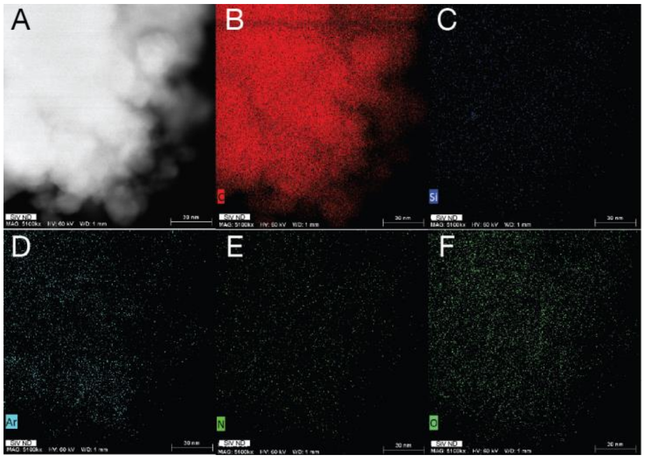


**Fig. S7. STEM-EDS composition maps.** Elemental mapping collected during STEM imaging (A) of carbon (B), silicon (C), argon (D), nitrogen (E), and oxygen (F). These maps reveal no evidence for aggregation or heterogeneous distribution of nitrogen or silicon dopants. However, the argon, while present throughout the nanodiamond, was localized in some pockets of the nanodiamond, suggesting incorporation via pore collapse and subsequent diamond growth in some instances. On the other hand, the low oxygen concentration is consistent with surface adsorbed oxygen present in other carbonaceous samples. (*6*, *60*).

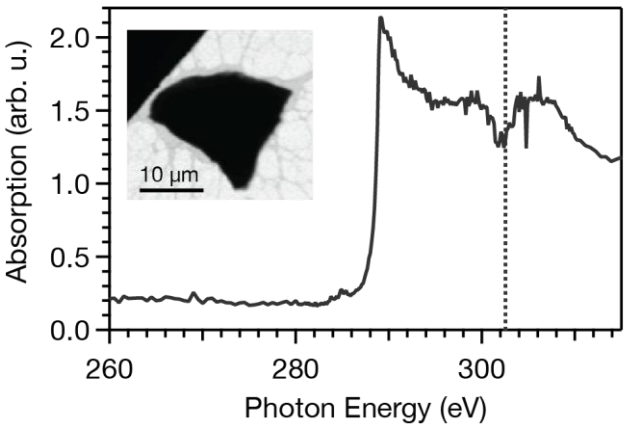


**Fig. S8. Carbon-K edge scanning transmission x-ray microscopy of nanodiamond synthesized from undoped carbon aerogel on a lacey carbon TEM grid.** Carbon absorption from STXM shows a strong $\sigma^*$ absorption and very little $\pi^*$. This demonstrates the high conversion to diamond and suggests that the dopants do not catalyze conversion from amorphous carbon to diamond. The dotted line at 302.5 eV points out the decrease in absorption due to the "second gap" in the electronic structure of diamond as discussed in the main text. Overall the carbon-K edge STXM absorption data almost perfectly mirror the EELS data in Fig. 2B. The inset shows the absorption intensity at 300 eV.

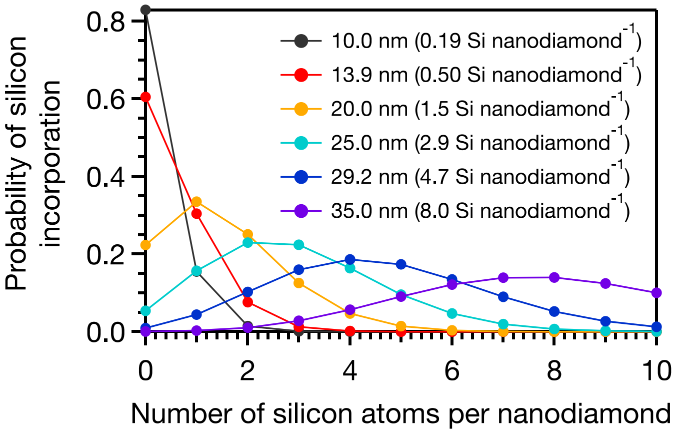


**Fig. S9. Poisson distribution of silicon incorporation per nanodiamond grain with varying size.** The probability that nanodiamond grains with varying diameter contain different number of

silicon atoms, using the experimental molar ratio of 4.5 $10^{-9}$ M, diamond density of 3.51 g $cm^{-3}$, and assuming spherical nanodiamonds with equal probability of silicon incorporation at every carbon lattice site. This analysis predicts that the experimentally observed nanodiamonds with average diameters of 14.0 nm and 29.2 nm have 0.50 and 4.7 silicon atoms per grain, respectively.

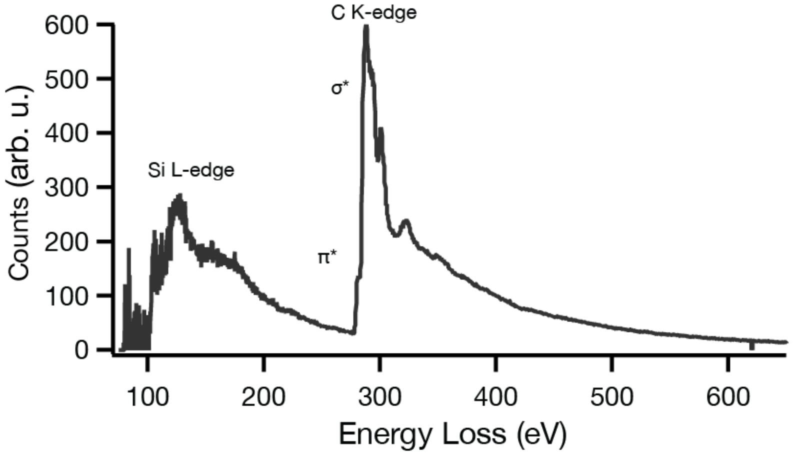


**Fig. S10. Integrated STEM-EEL spectrum image of the recovered silicon-doped carbon aerogel.** The EELS reveals the presence of silicon and carbon in the recovered nanodiamond. The carbon-K edge shows predominantly $sp^3$ carbon with a dip at 302.5 eV associated with the second gap of diamond and minimal $sp^2$ carbon, which is likely due to surface reconstruction.

**Table S1. Time-dependent DFT transition energies and oscillator strengths.** TD B3LYP/6-31G(d) transition energies, oscillator strengths and lists of the molecular orbitals which exhibit largest contributions to the absorption peak responsible for the ZPL for the $SiV^-$ containing nanodiamond with diameter of ~1.2 nm ($C_{119}SiH_{104}$) at both atmospheric and ~30GPa pressure. The contour plots for the corresponding molecular orbitals have been previously shown in Fig. 4 and S6.

| Atmospheric pressure | | |
|---|---|---|
| Transition Energy (eV) | Oscillator strength (arb.units) | MO mostly involved |
| 1.655 | 0.011 | HOMO-3 to LUMO |
| 1.736 | 0.050 | HOMO-2 to LUMO |
| 1.846 | 0.004 | HOMO-1 to LUMO |
| 29.6 GPa | | |
| 1.693 | 0.011 | HOMO-3 to LUMO |
| 1.783 | 0.051 | HOMO-2 to LUMO |
| 1.812 | 0.005 | HOMO-1 to LUMO |

**Table S2. Time-dependent DFT transition energies and oscillator strengths for ligand capping schemes.** The TD B3LYP/6-31g(d) transition energies, oscillator strengths, and the MO contributions for the systems which have replaced a surface hydrogen with either a carboxyl or hydroxyl group, pictured in fig. S11.

| Atmospheric pressure carboxyl ligand | | |
|---|---|---|
| Transition Energy (eV) | Oscillator strength (arb.units) | MO mostly involved |
| 1.655 | 0.011 | HOMO-3 to LUMO |
| 1.737 | 0.051 | HOMO-2 to LUMO |
| 1.838 | 0.004 | HOMO-1 to LUMO |
| Atmospheric pressure hydroxyl ligand | | |
| 1.640 | 0.010 | HOMO-3 to LUMO |
| 1.740 | 0.051 | HOMO-2 to LUMO |
| 1.835 | 0.005 | HOMO-1 to LUMO |

**Table S3. DFT orbital differences for the ligand capping schemes.** The HOMO-1, HOMO-2, HOMO-3 to LUMO energy averages for the diamonds with different capping ligands, pictured in fig. S11.

| Atmospheric Pressure | |
|---|---|
| System | Averaged HOMO-3, HOMO-2, HOMO-1 to LUMO MO average energy difference (eV) |

| | |
|---|---|
| Fully Hydrogen Passivated | 2.5589 |
| carboxyl | 2.5510 |
| hydroxyl | 2.5556 |

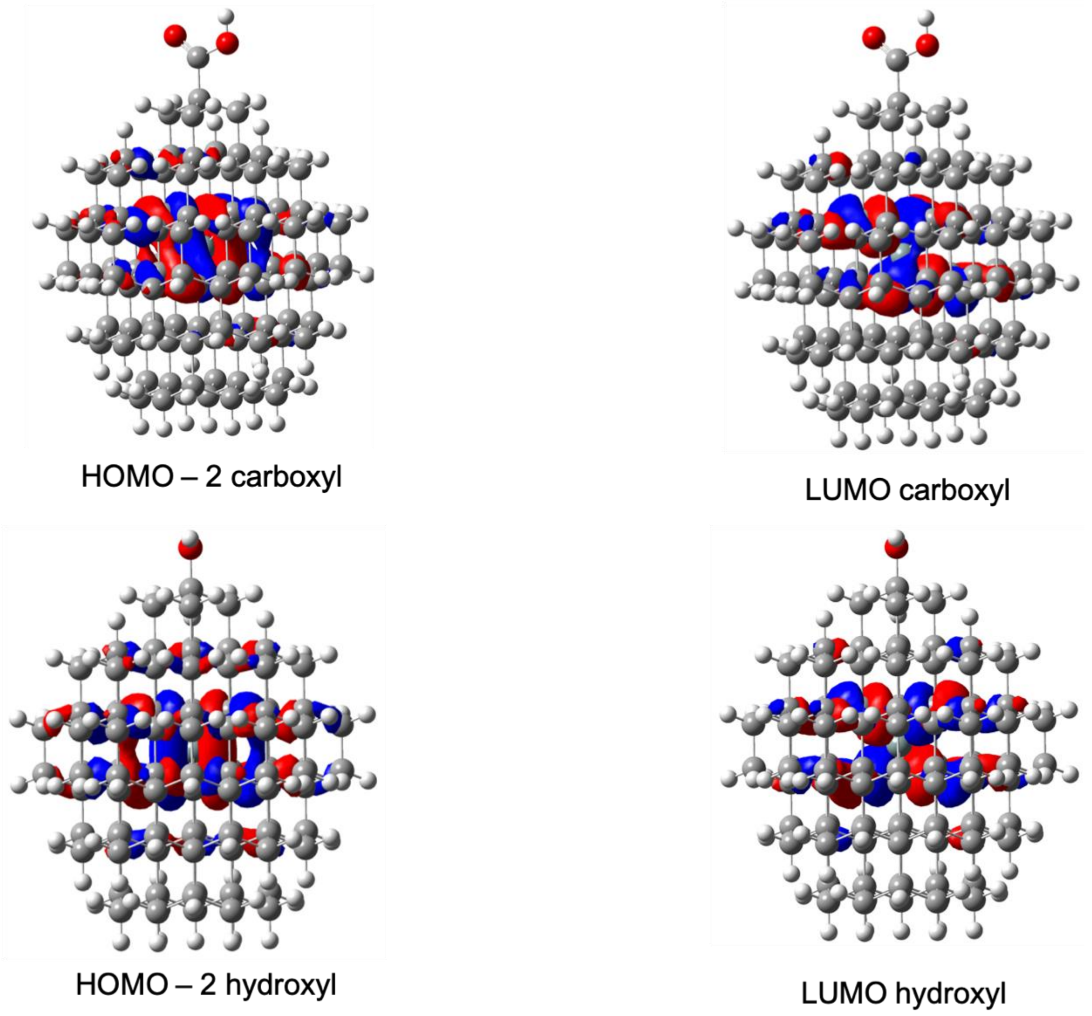


**Fig. S11. DFT modeling of surface capping scheme on SiV$^{-}$ center excitations.** The HOMO-2 and LUMO wavefunction surfaces for the HOMO-2 and the LUMO for the 1.2 nm diamonds with a carboxyl (-COOH) and hydroxyl (-OH) capping ligand.
